# Extent of Complete Retinal Pigment Epithelial and Outer Retinal Atrophy with Foveal Center Involvement is Associated with Visual Acuity

**DOI:** 10.1016/j.xops.2024.100612

**Published:** 2024-08-29

**Authors:** Norihiro Nagai, Hisashi Matsubara, Hiroto Terasaki, Takao Hirano, Aki Kato, Akiko Miki, Hiromasa Hirai, Fumiko Murao, Hiroko Imaizumi, Fumi Gomi, Yoshinori Mitamura, Nahoko Ogata, Sentaro Kusuhara, Tsutomu Yasukawa, Toshinori Murata, Taiji Sakamoto, Mineo Kondo, Hajime Shinoda, Yoko Ozawa

**Affiliations:** 1 Department of Ophthalmology, Keio University School of Medicine, Tokyo, Japan; 2 Department of Ophthalmology, Mie University Graduate School of Medicine, Tsu, Japan; 3 Department of Ophthalmology, Kagoshima University Graduate School of Medical and Dental Science, Kagoshima, Japan; 4 Department of Ophthalmology, Shinshu University School of Medicine, Matsumoto, Nagano, Japan; 5 Department of Ophthalmology and Visual Science, Nagoya City University Graduate School of Medical Sciences, Nagoya, Japan; 6 Division of Ophthalmology, Department of Surgery, Kobe University Graduate School of Medicine, Kobe, Japan; 7 Department of Ophthalmology, Nara Medical University, Nara, Japan; 8 Department of Ophthalmology, Tokushima University Graduate School, Tokushima, Japan; 9 Department of Ophthalmology, Sapporo City General Hospital, Sapporo, Japan; 10 Department of Ophthalmology, Hyogo Medical University, Nishinomiya, Japan; 11 Department of Clinical Regenerative Medicine, Fujita Medical Innovation Center Tokyo, Tokyo, Japan; 12 Eye Center, Fujita Health University Haneda Clinic, Tokyo, Japan

**Keywords:** Age-related macular degeneration, Geographic atrophy, Outer plexiform layer, Photoreceptor, Retinal pigment epithelium

## Abstract

**Purpose:**

To evaluate the OCT images of eyes with fovea-involved complete retinal pigment epithelial and outer retinal atrophy (cRORA) as well as best-corrected visual acuity (BCVA) to explore the pathogenesis of visual impairment and atrophy.

**Design:**

Retrospective observational study.

**Subjects:**

Data of eyes with cRORA associated with age-related macular degeneration with foveal center involvement were collected from 10 hospitals in Japan.

**Methods:**

Ophthalmic examination data, BCVA, and extents of retinal pigment epithelial and outer retinal atrophy (RORA), represented by choroidal hyper-transmission, and outer plexiform layer (OPL) deterioration, central retinal thickness (CRT), and central choroidal thickness (CCT) measured using built-in software on the sectional OCT images were evaluated.

**Main Outcome Measures:**

Relationship between BCVA and extents of RORA and OPL deterioration.

**Results:**

Of the 64 eyes of 64 patients (mean age: 76.8 ± 9.5 years old), 38 eyes (59.4%) belonged to men. Mean BCVA was 0.602 ± 0.475 (median: 0.523; range, −0.079 to 1.523) in logarithm of the minimum angle of resolution (logMAR). Mean extent of RORA was 2921 ± 1291 (median: 3172; range: 479–5985) μm. BCVA in logMAR positively correlated with extents of RORA (*P* = 0.004) and OPL deterioration (*P* = 0.004) and negatively correlated with CRT (*P* = 0.022). Best-corrected visual acuity ≥0.5 was associated with extents of RORA ≥3000 μm (odds ratio [OR], 4.227; 95% confidence interval [CI], 1.440–12.408; *P* = 0.009) and OPL deterioration ≥1700 μm (OR, 2.984; 95% CI, 1.034–8.609; *P* = 0.043), and presence of complete central outer plexiform layer defect (cCOD) (OR, 12.700; 95% CI, 2.439–66.132; *P* = 0.003), after adjusting for age and sex. The extent of RORA ≥3000 μm was associated with BCVA ≥0.5 (OR, 4.213; 95% CI, 1.437–12.356; *P* = 0.009), extent of OPL deterioration ≥1700 μm (OR, 58.682; 95% CI, 6.865–501.592; *P* < 0.001), and presence of cCOD (OR, 4.107; 95% CI, 1.339–12.604; *P* = 0.014), after adjusting for age and sex. The extent of RORA positively correlated with that of OPL deterioration (*P* < 0.001), CRT (*P* = 0.001), and CCT (*P* = 0.041).

**Conclusions:**

A longer extent of cRORA in the OCT images with foveal center involvement was associated with a longer extent of OPL deterioration and the presence of cCOD and worse BCVA. Further studies focusing on OPL changes are warranted for understanding the pathogenesis of RORA and vision loss.

**Financial Disclosures:**

Proprietary or commercial disclosure may be found in the Footnotes and Disclosures at the end of this article.

Age-related macular degeneration (AMD) is a leading cause of visual impairment in adults worldwide.^1^^,^^2^ Despite recent therapeutic interest, geographic atrophy (GA), an advanced AMD manifestation, lacks comprehensive understanding regarding its clinical characteristics and pathogenesis.

Geographic atrophy lesions are identifiable on blue or green light fundus autofluorescence (FAF) as well-demarcated areas of decreased signal intensity.^3^ However, because OCT is an essential imaging technology in clinical evaluation of the macula, the center of the retina, the Classification of Atrophy Meetings group members have developed a consensus terminology and criteria for defining atrophy based on OCT findings in AMD.^4^ They reported that photoreceptor atrophy can be detected before visible retinal pigment epithelium (RPE) atrophy appears, but in the later phase, RPE degeneration becomes apparent; this is now called retinal pigment epithelial and outer retinal atrophy (RORA). The main OCT finding of RORA is choroidal hyper-transmission based on photoreceptor degeneration and RPE attenuation or disruption. The atrophy gradually progresses, and complete retinal pigment epithelial and outer retinal atrophy (cRORA) is defined when hyper-transmission extends >250 μm in the OCT images.^4^^,^^5^ The use of OCT for evaluating GA has attracted attention and interest, with recent studies exploring automated diagnosis using artificial intelligence.^6^^,^^7^

Previous studies have revealed that lesions usually begin to appear in the extrafoveal area, subsequently expanding into the foveal center and toward peripheral direction.^8^, ^9^, ^10^ Although best-corrected visual acuity (BCVA) may be influenced by foveal center involvement, it was not necessarily associated with lesion localization or size in the natural course;^9^ the pathogenesis remained obscure.

In this study, we evaluated the OCT images of cRORA, which involved foveal center, and BCVA to explore the pathogenesis of RORA and resulting visual impairment.

## Methods

This study adhered to the tenets of the Declaration of Helsinki and was approved by the Ethics Committee of the Keio University School of Medicine (approved number, 20221111). The need for written informed consent was waived, and the opt-out approach was applied.

### Participants

This retrospective study enrolled patients with cRORA from 10 hospitals in Japan (Keio University Hospital, Mie University Hospital, Kagoshima University Hospital, Shinshu University Hospital, Nagoya City University Hospital, Kobe University Hospital, Nara Medical University Hospital, Tokushima University Hospital, Sapporo City General Hospital, and The Hospital of Hyogo College of Medicine). Complete retinal pigment epithelial and outer retinal atrophy was defined using criteria proposed by the Classification of Atrophy Meetings group; the criteria involved eyes with (1) a region of hyper-transmission ≥250 μm in diameter, (2) a zone of attenuation or disruption of the RPE ≥250 μm in diameter, (3) evidence of overlying photoreceptor degeneration, and (4) absence of scrolled RPE or other signs of an RPE tear.^4^ Patients with AMD-associated cRORA were included in the study. Patients with incomplete RORA with lesion sizes <250 μm, inherited diseases, retinal vascular occlusion, or fibrovascular pigment epithelial detachment were excluded. Patients with missing data were excluded. If both eyes were eligible, the eye with the greater extent of cRORA was included in the study. All eyes were of Japanese origin. The diagnosis of AMD was based on the guideline for diagnostic criteria for GA in Japan; the essential points included (1) at least 250 μm in diameter, (2) round／oval／cluster-like or geographic in shape, (3) sharp delineation, (4) hypopigmentation or depigmentation in the RPE, and (5) clearly visible choroidal large vessels.^11^^,^^12^

### Eye Examinations

Patients underwent ophthalmic examinations, including BCVA, fundus photography, spectral-domain OCT, and FAF imaging.

### OCT

**Figure 1:**
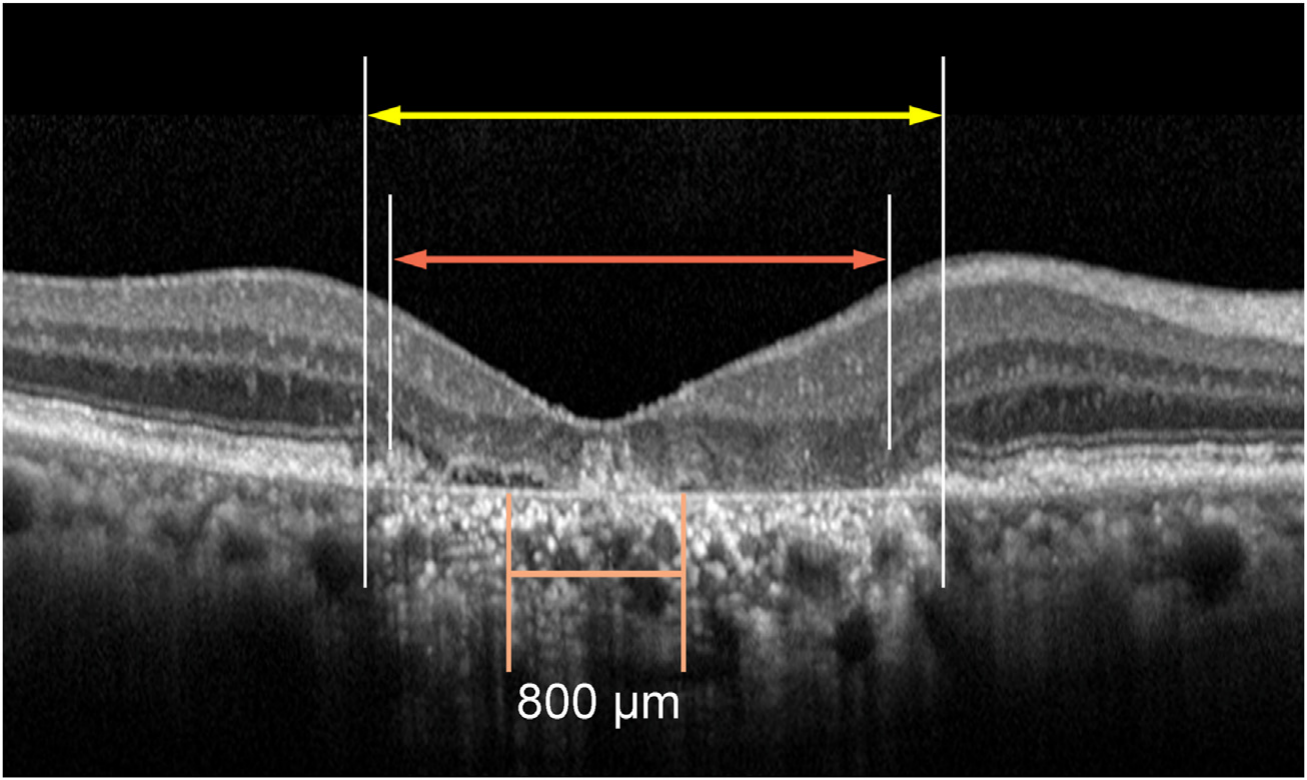
Measurement of extents of retinal pigment epithelial and outer retinal atrophy (yellow double arrows) and outer plexiform layer deterioration (orange double arrows) in foveal sections of OCT images The presence of complete central outer plexiform layer defect was defined by an absence of outer plexiform layer signals within 800 μm of diameter around the fovea.

OCT images obtained using a spectral-domain OCT system at each institute (Spectralis OCT; Heidelberg Engineering GmbH, Optovue: Visionix, or a Cirrus: Carl Zeiss Meditec, Inc) were analyzed. The OCT images passing through the fovea were obtained using a built-in protocol of line 30° scan (Spectralis OCT), 5-mm × 5-mm retina map scan (Optovue), and line 6-mm scan (Cirrus). Extents of RORA corresponding to that of hyper-transmission, and outer plexiform layer (OPL) deterioration judged by decreased intensity, discontinuity, and thinning of OPL finding within 6000 μm diameter from the fovea (Fig 1), central retinal thickness (CRT), and central choroidal thickness (CCT) were measured in OCT images of foveal scan using a built-in caliper and software of the device. The extent of RORA was measured both in the horizontal and vertical foveal sections, and the greater value was adopted. Outer plexiform layer findings were defined in the same section in which the extent of RORA was adopted. An OPL defect was analyzed in the foveal region and a complete central outer plexiform layer defect (cCOD) was defined by an absence of OPL signals within 800 μm diameter from the fovea where cone photoreceptor cells are concentrated; rod-free zone extends 350 to 720 μm in previous reports.^13^

### Statistical Analyses

IBM SPSS Statistics (version 29.0; IBM Corp) was used for all statistical analyses. Mann–Whitney *U* test, chi-square test, Pearson correlation coefficient analysis, univariate analyses, and multiple logistic regression were used to analyze the data. Statistical significance was set at *P* < 0.05. All data are presented as mean ± standard deviation values.

## Results

Of the 64 eyes of 64 patients who had cRORA, 38 eyes (59.4%) belonged to men, and the mean age of the patients was 76.8 ± 9.5 (range: 47–91) years old (Table 1). The mean BCVA was 0.602 ± 0.475, the median was 0.523, and the individual data ranged from −0.079 to 1.523 in logarithm of the minimum angle of resolution (logMAR). The mean extent of RORA was 2921 ± 1291 (median: 3172; range: 479–5985) μm, and involvement of the foveal center, defined by the deepest point of the foveal pit, was observed in all the eyes. Fifty-one eyes (79.7%) exhibited deterioration of OPL, where photoreceptor synapses transfer the signals to the secondary and/or intermediate neurons, and the mean deteriorated extent was 1647 ± 1313 (range: 0–4788) μm. Complete central outer plexiform layer defect, defined by an absence of OPL findings in the OCT images within 800 μm of diameter around the fovea, was found in 28 eyes (43.8%).

**Table 1:** Characteristics of the Eyes

Characteristic	Value
n	64
Age	76.8 ± 9.5 (79; 47-91)
Male	38 (59.4%)
BCVA in logMAR	0.602 ± 0.475 (0.523; −0.079 to 1.523)
Extent of RORA (μm)	2921 ± 1291 (3172; 479-5985)
Presence of OPL deterioration	51 (79.7%)
Extent of OPL deterioration (μm)	1647 ± 1313 (1413; 0-4788)
Presence of complete central OPL defect	28 (43.8%)
Central retinal thickness (μm)	113 ± 62 (108; 8-246)
Central choroidal thickness (μm)	169 ± 118 (141; 13-540)
Drusen	42 (65.6%)
Reticular pseudodrusen	19 (29.7%)
Hyperreflective foci	27 (42.2%)

BCVA = best-corrected visual acuity; logMAR = logarithm of the minimum angle of resolution; OPL = outer plexiform layer; RORA = retinal pigment epithelial and outer retinal atrophy. Data are presented as mean ± standard deviation (median; range).

Complete central OPL defect is defined by an absence of OPL within 800 μm diameter around the fovea.

Mean CRT and CCT were 113 ± 62 (median: 108; range: 8–246) μm and 169 ± 118 (median: 141; range: 13–540) μm, respectively. Drusen and reticular pseudodrusen were observed in 42 eyes (65.6%) and 19 eyes (29.7%), respectively, in the fundus photographs, whereas hyperreflective foci in the OCT images were in 27 eyes (42.2%).

**Figure 2:**
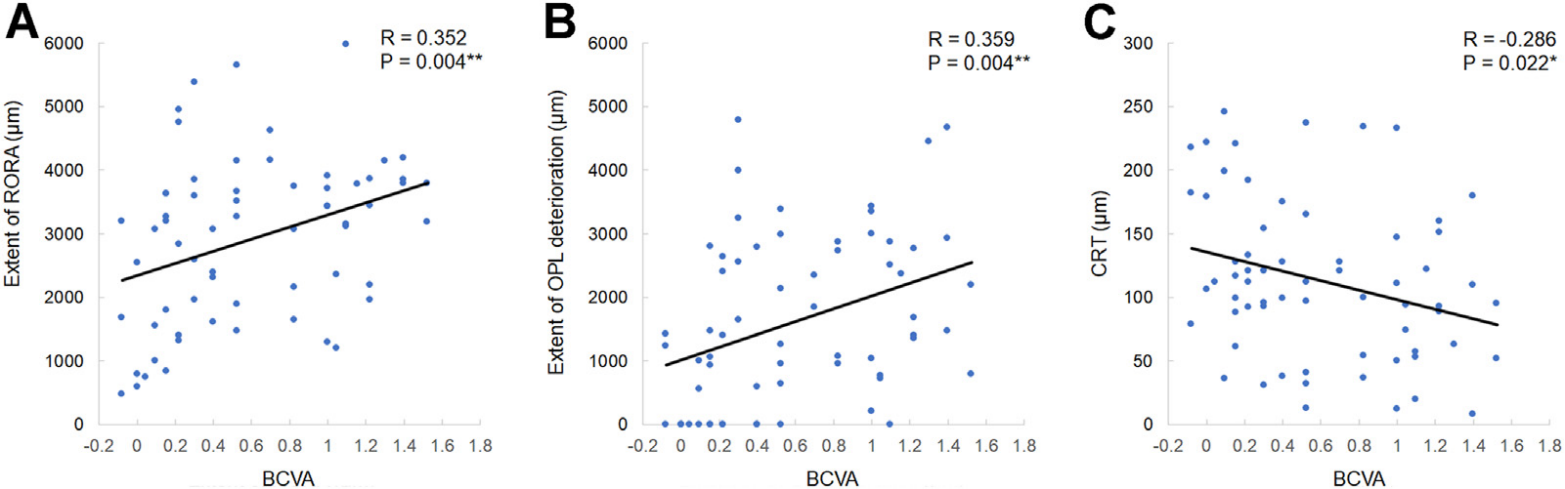
Correlations between best-corrected visual acuity and structural parameters measured in OCT images. Best-corrected visual acuity in logMAR positively correlated with the extent of retinal pigment epithelial and outer retinal atrophy (**A**) and extent of outer plexiform layer deterioration (**B**) and negatively correlated with central retinal thickness (**C**). Pearson correlation coefficient analysis. ∗*P* < 0.05 and ∗∗*P* < 0.01. BCVA = best-corrected visual acuity; CRT = central retinal thickness; logMAR = logarithm of the minimum angle of resolution; OPL = outer plexiform layer; RORA = retinal pigment epithelial and outer retinal atrophy.

In individual eyes, BCVA positively correlated with the extents of RORA (*P* = 0.004) (Fig 2A) and OPL deterioration (*P* = 0.004) (Fig 2B). Best-corrected visual acuity negatively correlated with CRT (*P* = 0.022) (Fig 2C).

The eyes with BCVA ≥0.5 in logMAR showed a longer mean extent of RORA (*P* = 0.005) (Table 2), and more frequently exhibited RORA ≥3000 μm (*P* = 0.011). The eyes with BCVA ≥0.5 also showed a longer mean extent of OPL deterioration (*P* = 0.005) and more frequently exhibited OPL deterioration ≥1700 μm (*P* = 0.049) and cCOD (*P* < 0.001). Mean CRT was significantly thinner in eyes with BCVA ≥0.5 in logMAR (*P* = 0.042).

**Table 2:** Comparison of the Eyes with BCVA ≥0.5 and <0.5 in logMAR

Characteristic	BCVA ≥0.5	BCVA <0.5	*P*
n	37	30	
Age	77.9 ± 9.9	75.5 ± 8.8	0.132
Male	20 (58.8%)	18 (60.0%)	1.000
Extent of RORA (μm)	3319 ± 1106	2469 ± 1314	0.005†
Extent of RORA ≥3000 μm	25 (73.5%)	12 (40.0%)	0.011∗
Extent of OPL deterioration (μm)	2056 ± 1147	1219 ± 1340	0.005†
Extent of OPL deterioration ≥1700 μm	19 (55.9%)	8 (26.7%)	0.049∗
Presence of complete central OPL defect	24 (70.6%)	4 (13.3%)	<0.001†
CRT (μm)	98 ± 62	129 ± 57	0.042∗
CRT <120 μm	23 (67.6%)	15 (50.0%)	0.335
CCT (μm)	167 ± 115	172 ± 118	0.803
CCT <170 μm	21 (61.8%)	19 (63.3%)	0.625
Drusen	19 (55.9%)	23 (76.7%)	0.115
Reticular pseudodrusen	9 (26.5%)	10 (33.3%)	0.593
Hyperreflective foci	15 (44.1%)	12 (40.0%)	0.803

BCVA = best-corrected visual acuity; CCT = central retinal thickness; CRT = central retinal thickness; logMAR = logarithm of the minimum angle of resolution; OPL = outer plexiform layer; RORA = retinal pigment epithelial and outer retinal atrophy.

Data are presented as mean ± standard deviation and number (%). Mann–Whitney *U* test and chi-square test. Complete central OPL defect is defined by an absence of OPL within 800 μm diameter around the fovea.

∗ *P* < 0.05.

† *P* < 0.01.

After adjusting for age and sex, extent of RORA ≥3000 μm (odds ratio [OR], 4.227; 95% confidence interval [CI], 1.440–12.408; *P* = 0.009) (Table 3), extent of OPL deterioration ≥1700 μm (OR, 2.984; 95% CI, 1.034–8.609; *P* = 0.043), as well as presence of cCOD (OR, 15.968, 95% CI, 4.293–59.391; *P* < 0.001) increased the risks for BCVA ≥0.5 in logMAR. The OR of the presence of visible drusen was 0.198 (95% CI, 0.051–0.768; *P* = 0.019), and it was a negative factor for exhibiting worse BCVA.

**Table 3:** Factors Associated with BCVA ≥0.5 in logMAR

Characteristic	Crude			Age, Sex-Adjusted Logistic Regression Analyses		
	OR	95% CI	*P*	OR	95% CI	*P*
Age	1.029	0.975–1.085	0.302			
Male	0.952	0.350–2.589	0.924			
Extent of RORA ≥3000 μm	4.167	1.450–11.972	0.008†	4.227	1.440–12.408	0.009†
Extent of OPL deterioration ≥1700 μm	3.094	1.080–8.865	0.035∗	2.984	1.034–8.609	0.043∗
Presence of complete central OPL defect	15.600	4.314–56.413	<0.001†	15.968	4.293–59.391	<0.001†
CRT <120 μm	2.091	0.758–5.764	0.386	2.056	0.739–5.719	0.168
CCT <170 μm	0.915	0.339–2.473	0.862	0.848	0.306–2.349	0.752
Drusen	0.386	0.130–1.139	0.085	0.198	0.051–0.768	0.019∗
Reticular pseudodrusen	0.720	0.246–2.111	0.549	0.655	0.218–1.965	0.450
Hyperreflective foci	1.184	0.437–3.206	0.739	1.033	0.366–2.918	0.950

BCVA = best-corrected visual acuity; CCT = central retinal thickness; CI = confidence interval; CRT = central retinal thickness; logMAR = logarithm of the minimum angle of resolution; OPL = outer plexiform layer; OR = odds ratio; RORA = retinal pigment epithelial and outer retinal atrophy.

Univariate analyses and multiple logistic regression analyses after adjusting for age and sex. Complete central OPL defect is defined by an absence of OPL within 800 μm diameter around the fovea.

∗ *P* < 0.05.

† *P* < 0.01.

Characteristics of the eyes with RORA ≥3000 μm were further analyzed; they exhibited worse mean BCVA (*P* = 0.005) (Table 4), consistent with the above data. Moreover, the mean extent of OPL deterioration was significantly longer (*P* < 0.001), and the extent of OPL deterioration ≥1700 μm (*P* < 0.001), and the presence of cCOD (*P* = 0.021) were more frequently observed compared with eyes with RORA <3000 μm. They also had a thinner mean CRT (*P* = 0.001) (in particular, CRT <120 μm [*P* = 0.004]) and had a thinner mean CCT (*P* = 0.046) and reticular pseudodrusen (*P* = 0.030).

**Table 4:** Comparison of the Eyes with Extent of RORA ≥3000 μm, and <3000 μm

Extent of RORA	≥3000 μm	<3000 μm	*P*
n	37	27	
Age	77.3 ± 7.9	76.0 ± 11.2	0.749
Male	21 (56.8%)	17 (63.0%)	0.797
BCVA in logMAR	0.742 ± 0.467	0.410 ± 0.404	0.005†
BCVA ≥0.5 in logMAR	25 (67.6%)	9 (33.3%)	0.011∗
Extent of OPL deterioration, μm	2400 ± 1182	673 ± 690	<0.001†
Extent of OPL deterioration ≥1700 μm	26 (70.3%)	1 (3.7%)	<0.001†
Presence of complete central OPL defect	21 (56.8%)	7 (25.9%)	0.021∗
CRT, μm	90 ± 51	144 ± 61	0.001†
CRT <120 μm	28 (75.7%)	10 (37.0%)	0.004†
CCT, μm	143 ± 102	205 ± 126	0.046∗
CCT <170 μm	26 (70.3%)	14 (51.9%)	0.912
Drusen	25 (67.6%)	17 (63.0%)	0.792
Reticular pseudodrusen	15 (40.5%)	4 (14.8%)	0.030∗
Hyperreflective foci	17 (45.9%)	10 (37.0%)	0.609

BCVA = best-corrected visual acuity; CCT = central retinal thickness; CRT = central retinal thickness; logMAR = logarithm of the minimum angle of resolution; OPL = outer plexiform layer; RORA = retinal pigment epithelial and outer retinal atrophy.

Data are presented as mean ± standard deviation and number (%). Mann–Whitney *U* test and chi-square test. Complete central OPL defect is defined by an absence of OPL within 800 μm diameter around the fovea.

∗ *P* < 0.05.

† *P* < 0.01.

Then, we analyzed the associated factors for exhibiting cRORA ≥3000 μm after adjusting for age and sex (Table 5). Not only BCVA ≥0.5 (OR, 4.213; 95% CI, 1.437–12.356; *P* = 0.009) but OPL deterioration ≥1700 μm (OR, 58.682; 95% CI, 6.865–501.592; *P* < 0.001) was strongly associated with cRORA ≥3000 μm; the presence of cCOD was also clearly associated (OR, 4.107; 95% CI, 1.339–12.604; *P* = 0.014). The CRT <120 μm (OR, 5.297; 95% CI, 1.771–15.844; *P* = 0.003) and the presence of reticular pseudodrusen (OR, 3.754; 95% CI, 1.062–13.274; *P* = 0.040) were also associated with cRORA ≥3000 μm.

**Table 5:** Factors Associated with the Eyes with Extent of Retinal Pigment Epithelial and Outer Retinal Atrophy ≥3000 μm

Characteristic	Crude			Age, Sex-Adjusted Logistic Regression Analyses		
	OR	95% CI	*P*	OR	95% CI	*P*
Age	1.014	0.962–1.069	0.604			
Male	0.588	0.210–1.646	0.312			
logMAR BCVA ≥0.5	4.167	1.450–11.972	0.008†	4.213	1.437–12.356	0.009†
Extent of OPL deterioration ≥1700 μm	54.167	6.550–447.933	<0.001†	58.682	6.865–501.592	<0.001†
Presence of complete central OPL defect	3.750	1.275–11.030	0.016∗	4.107	1.339–12.604	0.014∗
CRT <120 μm	5.289	1.790–15.630	0.003†	5.297	1.771–15.844	0.003†
CCT <170 μm	2.507	0.879–7.148	0.086	2.350	0.811–6.811	0.115
Drusen	0.923	0.324–2.629	0.881	0.728	0.224–2.366	0.597
Reticular pseudodrusen	3.920	1.125–13.660	0.032∗	3.754	1.062–13.274	0.040∗
Hyperreflective foci	1.445	0.524–3.983	0.477	0.468	0.468–3.869	0.582

BCVA = best-corrected visual acuity; CCT = central retinal thickness; CI = confidence interval; CRT = central retinal thickness; logMAR = logarithm of the minimum angle of resolution; OPL = outer plexiform layer; OR = odds ratio.

Univariate analyses and multiple logistic regression analyses after adjusting for age and sex. Complete central OPL defect is defined by an absence of OPL within 800 μm diameter around the fovea.

∗ *P* < 0.05.

† *P* < 0.01.

**Figure 3:**
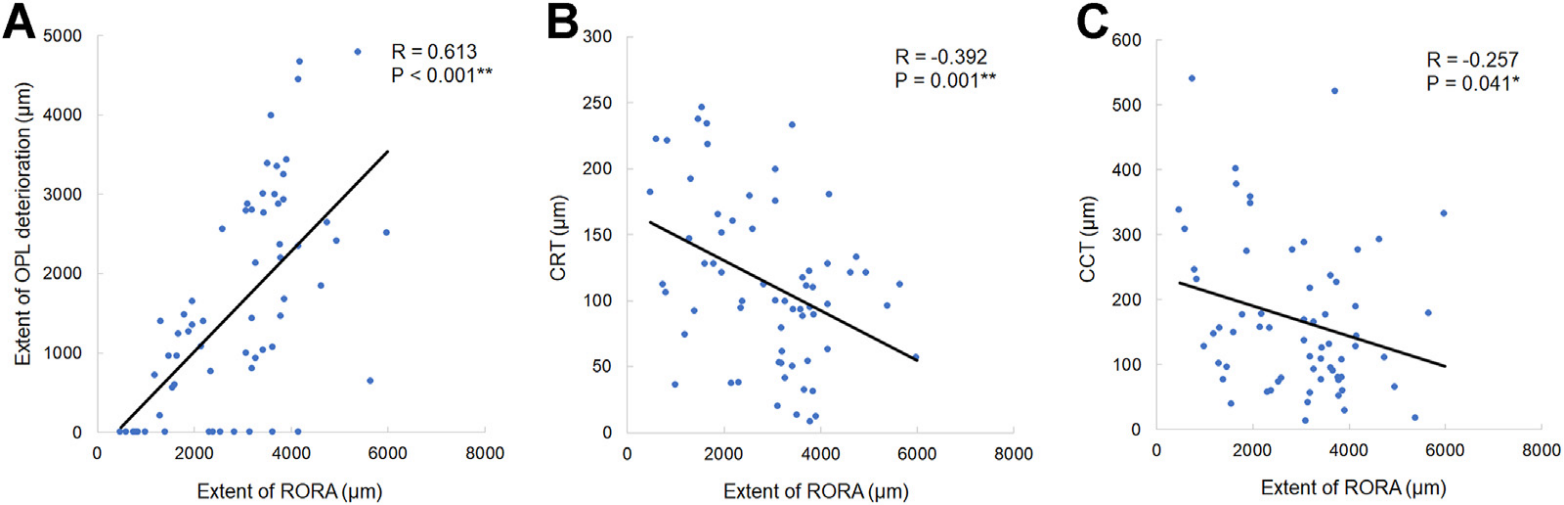
Correlations between the extent of RORA and other ocular parameters. The extent of RORA positively correlated with the extent of OPL deterioration (**A**) and negatively correlated with CRT (**B**) and CCT (**C**). Pearson correlation coefficient analysis. ∗*P* < 0.05 and ∗∗*P* < 0.01. CCT = central choroidal thickness; CRT = central retinal thickness; OPL = outer plexiform layer; RORA = retinal pigment epithelial and outer retinal atrophy.

The extent of RORA in individual eyes positively correlated with the extent of OPL deterioration (*P* < 0.001) (Fig 3A) and negatively correlated with CRT (*P* = 0.001) (Fig 3B) and CCT (*P* = 0.041) (Fig 3C).

Eyes with reticular pseudodrusen showed greater mean extent of RORA (*P* = 0.038), more frequently exhibited cRORA ≥3000 μm (*P* = 0.030), OPL deterioration ≥1700 μm (*P* = 0.026), and had thinner mean CCT compared with those without (*P* = 0.029) (Supplementary Table 1, available at www.ophthalmologyscience.org). Drusen was often observed in eyes with reticular pseudodrusen (*P* = 0.010) and hyperreflective foci (*P* = 0.007) (Supplementary Table 2, available at www.ophthalmologyscience.org). The presence of drusen was not associated with a greater extent of RORA (*P* = 0.794) (Supplementary Table 2, available at www.ophthalmologyscience.org).

## Discussion

We demonstrated that the BCVA of eyes with the foveal center involved cRORA positively correlated with the extents of RORA and OPL deterioration, and negatively correlated with CRT. Eyes with BCVA >0.5 in logMAR more often exhibited RORA ≥3000 μm, OPL deterioration ≥1700 μm, and cCOD after adjusting for age and sex. The RORA ≥3000 μm was associated with OPL deterioration ≥1700 μm, and the presence of cCOD after adjusting for age and sex. The extent of RORA positively correlated with the extent of OPL deterioration and negatively correlated with CRT and CCT.

The mean BCVA was 0.602 (median was 0.523) in logMAR, approximately corresponding to 0.25 (median: 0.3) in decimal and between 20/100 and 20/70 (median: 20/60) in Snellen charts. However, some eyes had a BCVA of −0.079 in logMAR (1.2 in decimal and better than 20/20 in the Snellen chart) and good visual acuity, whereas the area of cRORA involved the fovea in all eyes, as defined by the sectional OCT images. Therefore, photoreceptors, particularly cone photoreceptors, and RPE cells may not be completely absent in the area of cRORA. Given that rod photoreceptor cells are more vulnerable, as observed in early AMD where rods are affected before cones,^14^, ^15^, ^16^ cones may have remained functional, and BCVA was not necessarily worsened only by the presence of hyper-transmission in the fovea. The residual cone photoreceptors may be gradually lost in the later phase, which is at least partly related to the reduction in rods and their secretion of a neurotrophic factor, the rod-derived cone survival factor, which helps cone survival by promoting glucose uptake.^17^

In the current study, BCVA correlated with the extent of cRORA in individuals with foveal center involvement. A previous report has shown that the eyes with a longer extent of hyper-transmission are related to a significant increase in the number of locations with deep visual sensitivity defects ≤10 decibels in microperimetry; the RORA lesions with hyper-transmission ≥500 μm but not the lesions with hyper-transmission 250 to 499 μm, had significantly deeper visual sensitivity defects ≤10 decibels compared with the lesions with hyper-transmission <250 μm.^18^ Thus, enlargement of the RORA may be related to an increase in the area with low levels of visual sensitivity. Although the previous study involved eyes with RORA ≤1000 μm, given that the current study involved eyes with a relatively greater extent of RORA, deep visual sensitivity defects in the area of RORA may have appeared at a greater number of points, and involved the foveal region more frequently, and increased the risk of BCVA impairment.

The BCVA also correlated with the extent of OPL deterioration. We included not only OPL subsidence but also discontinuity and thinning of the OPL in the OCT sectional images to define OPL deterioration. This broader definition may involve milder OPL changes than subsidence changes. Given that the OPL is a layer composed of synapses between photoreceptors and secondary neurons and/or interneurons, deterioration reflects the dysfunction of the photoreceptor systems. Thus, it was natural that the cCOD, which represents substantial photoreceptor loss in the foveal region, was strongly associated with a worse BCVA.

**Figure 4:**
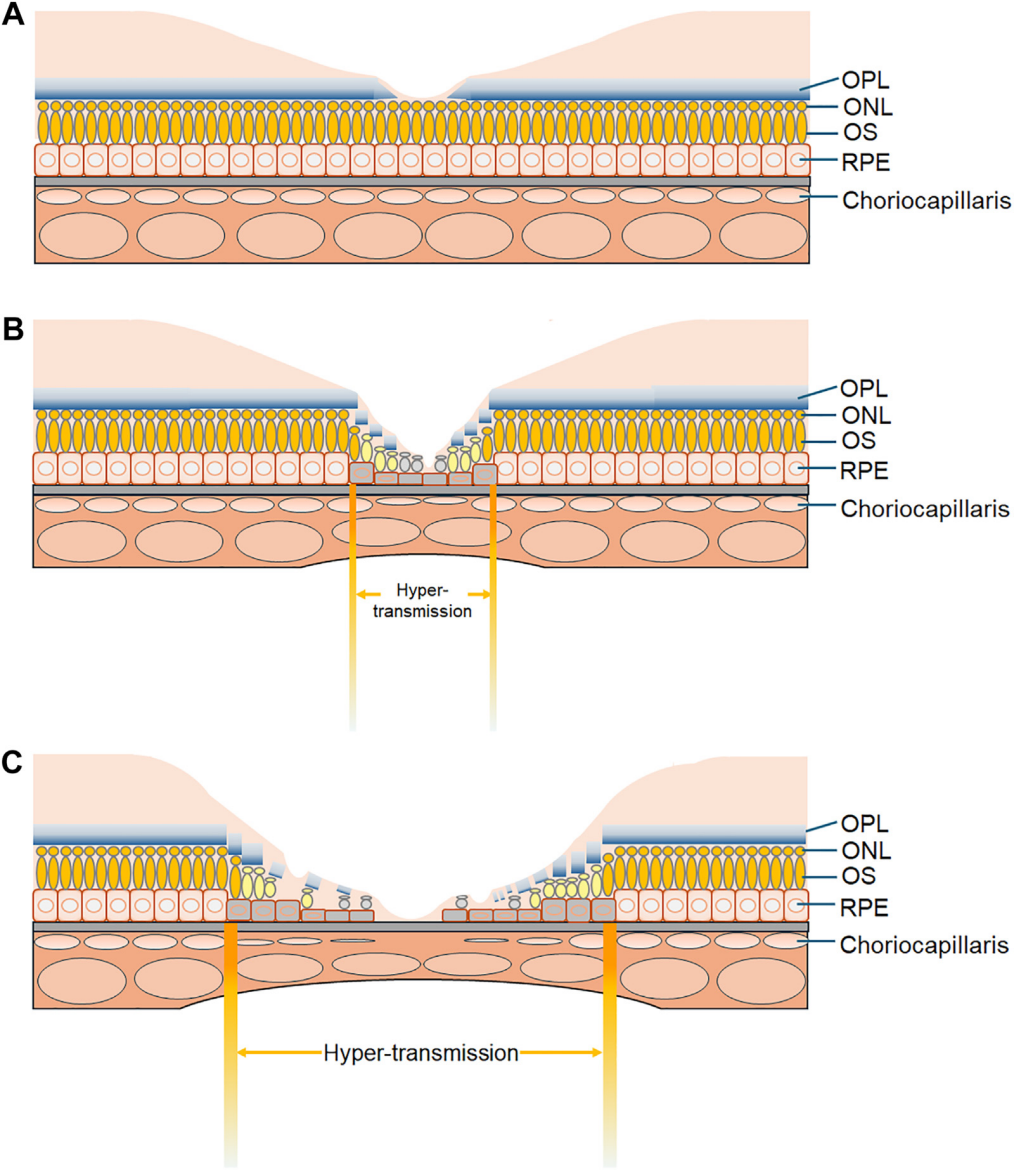
Hypothesis of the progression process of retinal pigment epithelial and outer retinal atrophy (RORA). (**A**) Healthy retina. (**B**) Relatively early phase after RORA was developed and the foveal region was involved. The OPL deterioration as a result of photoreceptor degeneration, appears in the area of RORA, and the hyper-transmission area. (**C**) Relatively later phase. The RORA extends in the horizontal and efferent direction, followed by the focal progression of photoreceptor degeneration which reduces photoreceptor layer thickness, and finally disrupts OPL; in the eyes with a greater extent of RORA, the risk of complete central OPL defect, and central visual loss increases. ONL = outer nuclear layer; OPL = outer plexiform layer; OS = outer segment; RPE = retinal pigment epithelium.

The extent of RORA correlated with the extent of OPL deterioration and the presence of cCOD. Eyes that exhibited a longer extent of RORA may have had a broader area of photoreceptor degeneration and central visual dysfunction. Outer plexiform layer deterioration was within the hyper-transmission area and the extent of OPL deterioration did not exceed the extent of hyper-transmission in most of the eyes, suggesting that the atrophy of some rows of the photoreceptors in the outer nuclear layer and the RPE may progress horizontally to enlarge the area of RORA after foveal center involvement,^10^^,^^19^ and, then, the atrophy of the certain local points of photoreceptors in the area of RORA may progress further reducing the number of photoreceptor rows, and outer nuclear layer thickness, to disrupt the OPL structure (Fig 4). Thus, atrophy progresses not only along the x-axes and y-axes of the retina, but also along the z-axis, thus, 3 dimensionally in the OCT images. In other words, the enlargement of the area with RORA may progress, followed by focal photoreceptor loss to deepen functional loss in the area of RORA, particularly in a relatively central area rather than the border of RORA. As a result, the frequency of cCOD was greater in the eyes with RORA ≥3000 μm, and the CRT was also negatively correlated with the extent of RORA in the current study.

Changes in OPL and cCOD may serve as biomarkers for photoreceptor degeneration. The cCOD was associated with RORA ≥3000 μm. Thus, the cCOD may be an advanced finding. Whether the suppression of atrophic area progression with drug treatment, such as by inhibitors for the complement cascade^20^, ^21^, ^22^ can also suppress the progression of focal photoreceptor loss represented by OPL changes, would be a future research interest.

A negative correlation was observed between the extent of RORA and CCT. Central choroidal thickness is reportedly reduced in the retinitis pigmentosa, in which the photoreceptors and RPE are affected.^23^^,^^24^ Alhtough RORA is observed focally, shorter CCT may represent whole choroidal thinning. Thus, choroidal change may not be the cause but could be a secondary effect of RORA. Considering that VEGF secreted by the RPE is indispensable for choroidal vasculature,^25^ local but severe RPE disorders may have caused an insufficient supply of VEGF and induced choroidal flow reduction. Alternatively, decreased oxygen and nutrient demands of the atrophic RPE and outer retina, where choroidal flow nourishes,^26^^,^^27^ may have reduced the choroidal thickness.

The presence of reticular pseudodrusen increased the risk of exhibiting greater RORA, consistent with a previous report.^10^ Drusen decreased the risk of worse BCVA in the current study. However, the presence of drusen may have been underestimated because it may have been involved in atrophic lesions by the time when the current study was conducted.

The current study has several limitations, including the relatively small sample size, being hospital-based and not population based. There was a possibility of eccentric fixation in BCVA measurements. The lesions were evaluated only by the OCT and not by the FAF, and the OCT models varied according to the hospital. Many patients with atrophic lesions may have stopped visiting the hospital because of the absence of treatment options for a long time. However, because new therapeutic approaches can be now available,^20^, ^21^, ^22^ experts may inform patients and general clinicians about potential therapies and ask them to visit the hospital and cooperate with the study to understand the pathogenesis more deeply. The use of OCT images rather than FAF images, in particular, the use of horizontal and vertical foveal scans, and not multiple scans, for the analyses, was attributable to the retrospective study design. The lesion area measured in FAF images or extrapolated using multiple OCT scans would be studied in the future. However, recording horizontal and vertical foveal OCT scans can be easily performed, and the current study results may be easily applied to daily clinical practices.

In conclusion, a longer extent of cRORA in the OCT images with foveal center involvement was associated with a longer extent of OPL deterioration and defect, particularly at the foveal region, and worse BCVA. The extents of RORA and OPL deterioration may serve as biomarkers for visual prognosis. The study will help understand the pathogenesis of RORA and GA and how to evaluate the lesion in a daily clinic. Further longitudinal studies are warranted to reveal whether the enlargement of the RORA from the central towards the peripheral retina is associated with advancing focal photoreceptor degeneration in the area of RORA. New therapeutic approaches would also be explored.
