# Supplementary material for: Extent of Complete Retinal Pigment Epithelial and Outer Retinal Atrophy with Foveal Center Involvement is Associated with Visual Acuity
Source: Ophthalmol Sci. 2024 Aug 29;5(1):100612. doi: 10.1016/j.xops.2024.100612 (PMC11832003; doi:10.1016/j.xops.2024.100612)
Supplement: Supplementary Table 1 [file mmc1.pdf]

Supplementary Table 1. Comparison of the eyes with reticular pseudodrusen (+) and reticular pseudodrusen (-) groups

	Reticular pseudodrusen (+)	Reticular pseudodrusen (-)	
n	19	45	
Age (y.o.)	78.7 ± 6.3	76.0 ± 10.4	0.409
Male	10 (52.6%)	28 (62.2%)	0.580
logMAR BCVA	0.619 ± 0.465	0.595 ± 0.473	0.837
logMAR BCVA≥ 0.5	9 (47.4%)	25 (55.6%)	0.593
Extent of RORA (μm)	3443 ± 966	2700 ± 1332	0.038*
Extent of RORA ≥ 3000 μm	15 (78.9%)	22 (48.9%)	0.030*
Extent of OPL deterioration (μm)	2173 ± 1434	1416 ± 1174	0.065
Extent of OPL deterioration ≥ 1700 μm	12 (63.2%)	14 (31.1%)	0.026*
Presence of central OPL defect	10 (52.6%)	18 (40.0%)	0.416
CRT (μm)	117 ± 60	111 ± 62	0.686
CRT < 120 μm	11 (57.9%)	27 (60.0%)	1.000
CCT (μm)	116 ± 62	192 ± 126	0.029*
CCT < 170 μm	16 (84.2)	24 (53.3%)	0.025*
Drusen	17 (89.5%)	25 (55.6%)	0.010*
Hyperreflective foci	12 (63.2%)	15 (33.3%)	0.051

Data are presented as mean ± standard deviation and number (%). Mann–Whitney U test and Chi-square test. BCVA, best-corrected visual acuity; RORA, Retinal Pigment Epithelial and Outer Retinal Atrophy; OPL, outer plexiform layer; CRT, central retinal thickness; CCT, central choroidal thickness; OPL, outer plexiform layer. Complete central OPL defect is defined by an absence of OPL within 800 μm diameter around the fovea. **P<0.01, *P<0.05.
