# Supplementary material for: Extent of Complete Retinal Pigment Epithelial and Outer Retinal Atrophy with Foveal Center Involvement is Associated with Visual Acuity
Source: Ophthalmol Sci. 2024 Aug 29;5(1):100612. doi: 10.1016/j.xops.2024.100612 (PMC11832003; doi:10.1016/j.xops.2024.100612)
Supplement: Supplementary Table 2 [file mmc2.pdf]

Supplementary Table 2. Comparison of the eyes with drusen (+) and drusen (-) groups

	Drusen (+)	Drusen (-)	P
n	42	22	
Age	79.7 ± 6.1	71.5 ± 11.7	0.007**
Male	23 (54.8%)	15 (68.2%)	0.422
logMAR	0.547 ± 0.453	0.734 ± 0.490	0.200
logMAR BCVA ≥ 0.5	19 (45.2%)	15 (68.2%)	0.114
Extent of RORA	2875 ± 1260	3016 ± 1286	0.794
Extent of RORA ≥ 3000 μm	24 (57.1%)	13 (59.1%)	1.000
Extent of OPL deterioration (μm)	1592 ± 1378	1830 ± 919	0.342
Extent of OPL deterioration ≥ 1700 μm	19 (45.2%)	8 (36.4%)	0.598
Complete central OPL defect	17 (40.5%)	11 (50.0%)	0.636
CRT (μm)	120 ± 57	101 ± 66	0.157
CRT < 120 μm	23 (54.8%)	15 (68.2%)	0.422
CCT (μm)	153 ± 87	196 ± 152	0.539
CCT < 170 μm	28 (66.7%)	12 (54.5%)	0.419
Reticular pseudodrusen	17 (40.5%)	2 (9.1%)	0.010*
Hyperreflective foci	23 (54.8%)	4 (18.2%)	0.007 **

Data are presented as mean ± standard deviation and number (%). Mann–Whitney U test and Chi-square test. RORA, Retinal Pigment Epithelial and Outer Retinal Atrophy; BCVA, best-corrected visual acuity; OPL, outer plexiform layer; CRT, central retinal thickness; CCT, central choroidal thickness. Complete central OPL defect is defined by an absence of OPL within 800 μm diameter around the fovea. **P<0.01, *P<0.05.
